# *Babesia* spp. in European wild ruminant species: parasite diversity and risk factors for infection

**DOI:** 10.1186/1297-9716-45-65

**Published:** 2014-06-13

**Authors:** Adam O Michel, Alexander Mathis, Marie-Pierre Ryser-Degiorgis

**Affiliations:** 1 Centre for Fish and Wildlife Health, Department of Infectious Diseases and Pathobiology, Vetsuisse Faculty, University of Bern, Bern, Switzerland; 2 Swiss National Centre for Vector Entomology, Institute of Parasitology, Vetsuisse Faculty, University of Zurich, Zurich, Switzerland

## Abstract

*Babesia* are tick-borne parasites that are increasingly considered as a threat to animal and public health. We aimed to assess the role of European free-ranging wild ruminants as maintenance mammalian hosts for *Babesia* species and to determine risk factors for infection. EDTA blood was collected from 222 roe deer (*Capreolus c. capreolus*), 231 red deer (*Cervus e. elaphus*), 267 Alpine chamois (*Rupicapra r. rupicapra*) and 264 Alpine ibex (*Capra i. ibex)* from all over Switzerland and analysed by PCR with pan-*Babesia* primers targeting the 18S rRNA gene, primers specific for *B. capreoli* and *Babesia* sp. EU1, and by sequencing. *Babesia* species, including *B. divergens, B. capreoli, Babesia* sp. EU1, *Babesia* sp. CH1 and *B. motasi*, were detected in 10.7% of all samples. Five individuals were co-infected with two *Babesia* species. Infection with specific *Babesia* varied widely between host species. Cervidae were significantly more infected with *Babesia* spp. than Caprinae. *Babesia capreoli* and *Babesia* sp. EU1 were mostly found in roe deer (prevalences 17.1% and 7.7%, respectively) and *B. divergens* and *Babesia* sp. CH1 only in red deer. Factors significantly associated with infection were low altitude and young age. Identification of *Babesia* sp. CH1 in red deer, co-infection with multiple *Babesia* species and infection of wild Caprinae with *B. motasi* and *Babesia* sp. EU1 are novel findings. We propose wild Caprinae as spillover or accidental hosts for *Babesia* species but wild Cervidae as mammalian reservoir hosts for *B. capreoli*, possibly *Babesia* sp. EU1 and *Babesia* sp. CH1, whereas their role regarding *B. divergens* is more elusive.

## Introduction

Babesiosis is a tick-borne disease caused by protozoan parasites of the genus *Babesia* and affecting a wide range of domestic and wild mammalian hosts. Disease signs vary in severity from silent infection to acute circulatory shock with anemia, depending on susceptibility, immunity and age of the host, and on *Babesia* species and parasite load [1-3]. Worldwide, *Babesia* species are primarily of veterinary importance [1,4] but human cases mainly reported from North America and Europe have raised the question of whether they may also be emerging human pathogens [5].

In Europe, three *Babesia* species are of particular interest in ruminants: *B. divergens, B. capreoli* and *Babesia* sp. EU1 (also known as *B. venatorum *[6]). *Babesia divergens* is the principal agent of babesiosis in cattle [7]. It is capable of infecting gerbils (*Meriones unguiculatus*), sheep (*Ovis aries*) and reindeer (*Rangifer t. tarandus*) [8-10] and was reported in single cases as causative agent of fatal disease in immunosuppressed or splenectomized humans [5]. *Babesia capreoli* is not known to be pathogenic for humans or livestock [11,12] but is prevalent in free-ranging asymptomatic roe deer (*Capreolus c. capreolus) *[13,14] and occasionally causes disease in wild Caprinae [15]. *Babesia* sp. EU1 was first identified in 2007 in a human patient from Germany who displayed associated clinical symptoms [16]. Since then, the parasite has been reported in free-ranging roe deer in many European countries including France [17], Germany [18], Slovenia [19], Spain [20] and Poland [21]. Additionally, *B. bigemina* and *B. bovis* were identified as the cause of babesiosis outbreaks in cattle [22,23]; subclinical infections with *B. motasi* were reported in small domestic ruminants such as goat and sheep [24]; and a new species of *Babesia* tentatively described as *Babesia* sp. CH1 was found in ticks feeding on red deer from Switzerland [25].

Clinical babesiosis in free-ranging wild ruminants appears to be rare. Documented cases concern only Caprinae and were caused by either *B. capreoli* or *B. ovis *[15,26]. In contrast, numerous studies in Europe and abroad have documented the occurrence of silent *Babesia* spp. infections in free-ranging cervids [3]. However, there has been confusion regarding the identity of the detected *Babesia* species, particularly in roe deer. While *Babesia* from roe deer were formerly referred to as *B. divergens* or *B. divergens*-like, recent investigations showed that roe deer are usually infected with *B. capreoli*, which is antigenically and morphologically indistinguishable from *B. divergens *[12]. Differences between the 18S rRNA gene of *B. divergens* and *B. capreoli* have been described at only three positions namely 631, 663 and 1637, with AAC for *B. divergens* and GTT for *B. capreoli *[11]; further, differences in the sequences of the internal transcribed spacers 1 and 2 (ITS1, ITS2) have been reported [27]. Relying on nucleotide identities at positions 631 and 663 of the 18S rRNA gene, the only confirmed infections with *B. divergens* in free-ranging wild ruminants so far were in red deer from Ireland [28] and more recently in two roe deer from Poland [21].

In Switzerland, occasional outbreaks of babesiosis caused by *B. divergens* have been reported in cattle only [29] but this parasite has been identified in ticks (*Ixodes ricinus)* collected from both domestic cattle and free-ranging red deer (*Cervus e. elaphus*) [25]. Between 2005 and 2006, five Alpine chamois succumbed to babesiosis due to *B. capreoli *[15,27]. Subsequent investigations in the two affected Swiss regions tentatively identified roe deer and red deer as potential reservoir for *B. capreoli*[30]. In that study, however, detected *Babesia* were not systematically identified to species level, leaving the possibility of prevalence errors. Furthermore, sample size was limited, especially for red deer.

Overall, despite increasing numbers of studies on *Babesia* spp. in wildlife, gaps of knowledge remain regarding the spectrum of parasite species infecting wild hosts, the potential role of wildlife populations as source of infection for livestock and humans, and the epidemiology of *B. capreoli*, which causes babesiosis in Alpine chamois. To address these questions, we carried out a country-wide survey in free-ranging indigenous wild ruminants in Switzerland. This country is of particular interest for such a study as it is characterized by various landscapes, climatic patterns and vegetation coverage possibly influencing parasite occurrence (i.e. tick and mammal host occurrence); it also hosts large numbers of four important European wild ruminant species, namely roe deer, red deer, Alpine chamois, and Alpine ibex (*Capra ibex ibex*). The specific objectives of the study were (1) to document the occurrence and diversity of *Babesia* species in free-ranging populations of wild ruminants, including the identification of potentially yet undescribed species of *Babesia*; and (2) to assess risk factors for infection.

## Materials and methods

### Study area

The study area covered the whole territory of Switzerland (41 285 km^2^), except for the canton of Bern (5659 km^2^), which did not participate in the sampling campaign due to administrative constraints. Switzerland can be divided into four main bioregions (Jura, Plateau, Alps and South), which differ largely in climate and geographical features [31]. The four wild ruminant species endemic to Switzerland (roe deer, red deer, Alpine chamois and Alpine ibex, with an estimated population size ranging from approximately 15 600 for ibex to 113 000 for roe deer [32]) show a nonhomogeneous distribution, reflecting the suitability of the landscape as species-specific habitat. The small, introduced populations of Sika deer (*Cervus nippon*; ca. 250–300 individuals in northern Switzerland) [33] and mufflon (*Ovis aries orientalis*; ca. 200–300 in south-western Switzerland) [34] were not considered in our study.

### Animals and samples

Blood samples from 984 wild ruminants (222 roe deer, 231 red deer, 267 chamois and 264 ibex) collected from September 2009 to January 2010 in the framework of a cross-sectional study on virus infections [32,35] were used for the present study. Blood was mostly collected from animals hunted, culled or found dead and was sampled by game wardens or hunters using standard sampling kits containing gloves, sterile EDTA tubes and a syringe. We also used samples from five carcasses submitted for post-mortem investigation to the Centre for Fish and Wildlife Health (FIWI) in Bern, Switzerland, and from six live animals captured in the framework of ecological studies. Additionally, we included samples from eight chamois submitted to the FIWI between 2005–2009, confirmed to be infected with *B. capreoli* and which had died of hemolytic anemia (i.e., five cases previously reported [15] and three more recent cases from 2009). Blood samples from dead animals were collected either directly from the heart or from the body cavities. Samples from live animals were collected by puncturing the jugular vein during chemical immobilization, with the authorizations of competent authorities (see [32]).

Immediately after collection (sampling at the laboratory) or upon receipt (sampling in the field), samples were transferred to 1.5 mL Eppendorf tubes and frozen at -20 °C until further use. Table 1 compiles the demographic and geographic data obtained for each animal by means of a data sheet completed by the submitter.

**Table 1:** **Demographic data of the animals tested for *Babesia *infection**

**Demographic data**	**Species**			
	**Roe deer (*n*** **= 222)**	**Red deer (*n*** **= 231)**	**Alpine chamois (*n*** **= 267)**	**Alpine ibex (*n*** **= 264)**
< 1-year	62	61	7	1
≥ 1-year	159	169	258	260
Age unknown	1	1	2	3
Female	117	107	119	129
Male	105	119	145	134
Sex unknown	0	5	3	1
Mean altitude	860	1198.4	1649.5	2321.5
St. deviation	± 418.1	± 455.7	± 653.1	± 408.6
Altitude range	355-2439	245-2407	431-2909	442-2712

Altitudinal mean and range are both given in meters above sea level (m.a.s.l.).

### Laboratory analysis

DNA was extracted from aliquots of whole EDTA blood using the DNeasy blood & tissue kit (Qiagen, Hombrechtikon, Switzerland). Analyses were carried out according to the manufacturer’s standard protocol except for the blood quantity and initial incubation step. Due to severe hemolysis or coagulation of some blood samples, sample volume was decreased and incubation period with proteinase K was extended to increase the final DNA concentration. More specifically, 100 μL of EDTA blood were incubated overnight (instead of 200 μL incubated for 15 min) at 56 °C with 20 μL of proteinase K, 100 μL phosphate buffered saline and 200 μL of buffer AL. In a final step of purification, DNA was eluted in 100 μL buffer AE and stored at -20 °C until further use.

DNA was amplified by PCR in 100 μL assays prepared as previously described [27] but with 20 μL of DNA sample instead of 25 μL. Table 2 describes primer specifications and PCR cycling conditions. Initially, all samples were screened for *Babesia* spp. using the pan-*Babesia* primers BabF/R. Positive samples were then screened for *Babesia* sp. EU1. Samples positive in the pan-*Babesia* PCR and either positive or negative for *Babesia* sp. EU1 were also screened for *B. capreoli.* This was performed using the newly designed specific primers described in Table 2.

**Table 2:** Primer sequences and PCR conditions used in this study

**Primer designation**	**Specificity**	**Locus**	**Sequence (5’-3’)**	**Fragment size**	**Annealing temp (°C)**	**Extension time (s)**	**No. cycles**	**Reference**
BabsppF1	*Babesia* spp.*	18S rRNA gene	GTTTCTGMCCCATCAGCTTGAC	422-440	61	45	40	[25]
BabsppR			CAAGACAAAAGTCTGCTTGAAAC					
BabcapF	*Babesia capreoli*	rRNA locus (ITS2)	AGGAACCACACTTTTACTGGTTT	210	62	30	40	This study
BabcapR			CATCCACTTGCYATAGAAATACAA					
BabsppF1	*Babesia* sp. EU1	18S rRNA gene	GTTTCTGMCCCATCAGCTTGAC	362	61	45	40	[25]
BabEU1			AGACAAGAGTCAATAACTCGATAAC					

*Bovine *Babesia* spp*.*: *B. bigemina, B. capreoli, B. canis, B. crassa, B. divergens, B. major, B. motasi, B. odocoilei, B. ovata, Babesia* sp. EU1.

DNA samples positive to the pan-*Babesia* primers but negative to both BabF/EU1R and BabcapF1/R were sequenced. Amplicons obtained from amplification with the pan-*Babesia* primers were purified using the Qiaquick PCR purification kit (Qiagen) following the manufacturer’s instructions. Purified products were sent for sequencing to Synergene Biotech GmbH (Schlieren, Switzerland). Phylogenetic analyses were conducted using BioNumerics 7 (Applied-Maths NV, Austin, Texas, USA [36]). We constructed a Neighbour-Joining tree with reliability tested using bootstrapping with 1000 pseudoreplicates.

### Data management and statistics

Data coding and management was done in MS Excel and OpenOffice spreadsheets. Statistical analyses were performed with the NCSS 2007 software (Hintze J., 2006; NCSS, Kaysville, Utah, USA [37]). Prevalences were calculated with an assumed test sensitivity and specificity of 100% (considering the combined results of the pan-*Babesia* PCR, both specific PCRs and sequencing). Chamois diagnosed with clinical babesiosis were not included in the prevalence calculations because they had not been submitted in the frame of the survey.

We computed a Fisher’s exact test (FET) to assess associations between prevalence of infection with different *Babesia* species and potential risk factors for infection such as host species, sex, age, sampling unit, and cause of death (hunted/culled for humane or population control reasons vs. found dead). The Mann–Whitney U test was applied for comparisons of altitudes. Significance level for all tests was set at *p* < 0.05. Statistical significance of differences was not assessed for parasite/host combinations with very low prevalence (chamois and ibex for *B. capreoli, Babesia* sp. EU1 and *B. motasi*; red deer for *B. divergens*). For the association between sampling unit and prevalence of infection, sampling units with a sample size of less than 10 individuals were not included. For spatial representation and mapping we used QGIS software [38].

## Results

### *Babesia* diversity

Of 984 tested individuals, 105 (10.7%) tested positive with pan-*Babesia* primers, and five different *Babesia* species could be identified by specific PCRs or sequencing. An overview of the identified *Babesia* species and number of infected animals is given in Table 3. *Babesia capreoli* was the most commonly identified species in this study, followed by *Babesia* sp. EU1, *Babesia* sp. CH1, *B. divergens* and *B. motasi*. Co-infection with *B. capreoli* and *Babesia* sp. EU1 was identified in three roe deer and two chamois.

**Table 3:** **Prevalences of the different *Babesia *species identified in four species of wild ruminants**

	**Roe deer (*n*** **= 222)**		**Red deer (*n*** **= 231)**		**Alpine chamois (*n*** **= 267)**		**Alpine ibex (*n*** **= 264)**	
	**No. infected**	**Prevalence (95% CI)**	**No. infected**	**Prevalence (95% CI)**	**No. infected**	**Prevalence (95% CI)**	**No. infected**	**Prevalence (95% CI)**
*Babesia* spp.	53	23.9% (18.4-30.0)	40	17.3% (12.7-22.8)	8	3.0% (1.3-5.8)	4	1.5% (0.4-3.8)
*B. capreoli*	38	17.1% (12.4-22.7)			2	0.8% (0.1-2.7)		
*B. divergens*			6	2.6% (1.0-5.6)				
*Babesia* sp. EU1	17	7.7% (4.5-12.0)			7	2.6% (1.1-5.3)	1	0.38% (0.01-2.1)
*Babesia* sp. CH1			11	4.8% (2.4-8.4)				
*B. motasi*					1	0.4% (0.01-2.07)	3	1.1% (0.2-3.3)

Prevalence is calculated as the number of infected individuals over the total number of individuals tested within the same wild ruminant species. The 95% confidence interval (95% CI) is given in brackets beside the prevalence. Prevalence for *Babesia* spp. includes all positive individuals with the pan-*Babesia* PCR. Prevalences for the different *Babesia* species were calculated based on results of the specific PCRs or sequencing (incomplete sequences from one roe deer and 23 red deer excluded). Co-infected individuals with *B. capreoli/Babesia* sp. EU1 (three roe deer and two chamois) were considered once for each *Babesia* species.

Roe deer had the highest prevalence for *Babesia* spp. with *B. capreoli* being identified in 17.1% of the animals. In twelve of these 38 roe deer, PCRs using the *B. capreoli* specific primers Babcap F/R gave negative results, and species identification was achieved by sequencing the pan-*Babesia* amplicons, revealing 100% identity to the *B. capreoli* reference sequence from France BAB1220 [GenBank: AY726009]. For verification purposes, two amplicons from samples that were positive with *B. capreoli* and one sample that was positive with *Babesia* sp. EU1 specific primers were sequenced at the 18S rRNA gene (using pan-*Babesia* primers) revealing 100% identities with reference sequences (GenBank: AY726009 and GenBank: DQ312434, respectively). One roe deer amplicon could not be characterized due to poor sequence quality.

In red deer, the amplicons of six samples were identified as *B. divergens* of bovine origin [GenBank: AY046576], and 11 as *Babesia* sp. CH1 [Genbank: DQ312432]. Amplicons from 23 red deer were only identifiable to genus level due to insufficient sequence quality (weak signal strength, short segment reads or unclear nucleotide designation).

*Babesia* prevalences were low in Alpine chamois and Alpine ibex, and *B. motasi* [GenBank: AY260180] was identified in four animals.

**Figure 1:**
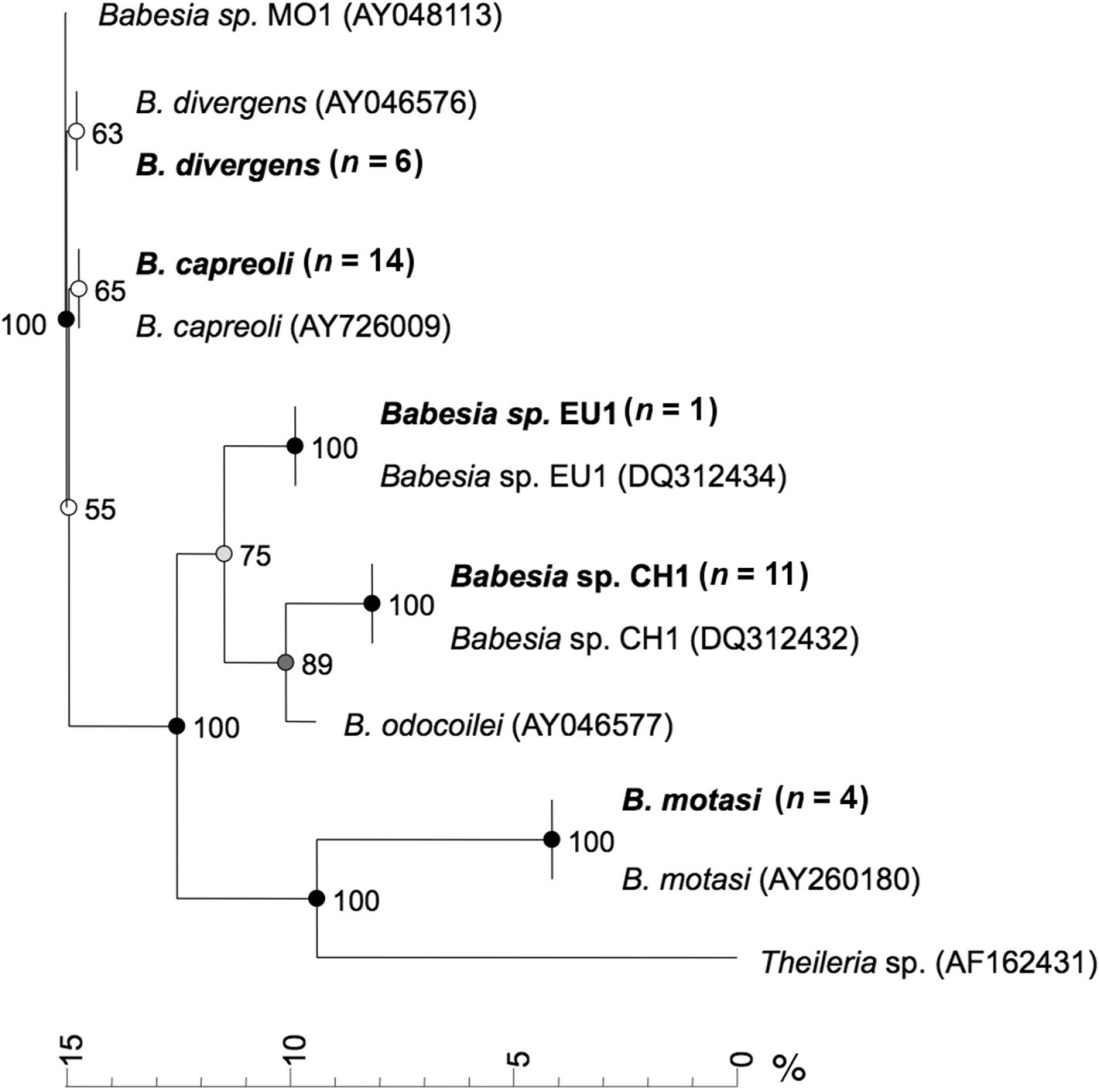
**Neighborhood joining tree of partial *Babesia *18S rRNA gene sequences.** Sequences from wild ruminants from the present study are highlighted in bold (number of identical sequences in brackets). Selected reference piroplasm sequences from GenBank (accession numbers in brackets) are also shown. Bootstrap values indicated at each node base are on N = 1000 replicates. Bar = percentage of difference between sequences.

All sequences of *B. divergens* analysed in this study form a clade separated from the one made of *B. capreoli* sequences, supported by 65% bootstrapping (Figure 1). Sequences of *Babesia* sp. EU1 cluster with reference sequences to which they are identical, as do *B. motasi* sequences. Babesia sp. CH1 from red deer is clearly separated from *B. odocoilei* (supported by 89% bootstrapping), which is the closest known *Babesia* species (Figure 1).

### Risk factors for infection

#### ***Host species***

Prevalence of infection with *Babesia* spp. did not significantly differ within Cervidae, i.e. between roe deer and red deer (*p* = 0.103), or within Caprinae, i.e. between ibex and chamois (*p* = 0.382). In contrast, there was a significant difference between Cervidae and Caprinae (*p* < 0.0001).

Prevalence of infection with specific *Babesia* varied widely among host species (Table 3). *Babesia capreoli* was detected more often in roe deer (17.1%) than chamois (*n* = 2, 0.8%, *p* < 0.0001) and in none of the tested red deer and ibex (*p* < 0.0001 and *p* < 0.0001, respectively). Similarily, *Babesia* sp. EU1 was found more frequently in roe deer (7.7%) than chamois (*n* = 7, 2.6%, *p* = 0.01) and was found in only one ibex (0.4%, *p* < 0.0001) and none of the red deer (*p* < 0.0001). Co-infections with *B. capreoli* and *Babesia* sp. EU1 were detected in three roe deer and two chamois. *Babesia divergens* and *Babesia* sp. CH1 were only detected in red deer, and *B. motasi* was identified only in chamois and ibex.

#### ***Sex***

We found no relationship between sex and *Babesia* infection, both when *Babesia* spp. and all species of ruminants were considered together (*P* = 0.406) and when *Babesia* species and host species were looked at independently.

#### ***Age***

There was a significant association between young age and infection with *Babesia* spp. in roe deer. Twenty-three of 62 (37.1%) roe deer kids (< 1-year old) were infected with *Babesia* spp. as opposed to 29 of 159 (18.2%) roe deer that were 1-year or older (*p* = 0.00451). This was also observed when different *Babesia* species were considered separately (*B. capreoli*, *p* = 0.0148; *Babesia* sp. EU1, *p* = 0.0246). Furthermore, four out of the five individuals showing concurrent infections with both *B. capreoli* and *Babesia* sp. EU1 were less than 1 year of age.

#### ***Altitude***

Regardless of the infection status, mean altitudes of sampling sites significantly differed among wild ruminant species (*p* < 0.0001). Cervidae were found at significantly lower altitudes ($\bar{x}=1031.5$ m.a.s.l., SD = 466.7) than Caprinae (μ = 1972.5 m.a.s.l., SD = 645.4; *p* < 0.0001). All host species combined, individuals positive for *Babesia* spp. were found at significantly lower altitudes ($\bar{x}=893.8$, SD = 485.6) than individuals that were not ($\bar{x}=1616.4$, SD = 725.5; *p* < 0.0001). This altitudinal difference was also observed when each host species was analysed independently. Roe deer positive for *B. capreoli* were sampled at significantly lower altitudes (μ = 682.9 m.a.s.l., SD = 258.4) than negative individuals (μ = 897.3 m.a.s.l., SD = 434.6; *p* = 0.0036). Similarly, the mean altitude of red deer positive for *Babesia* sp. CH1 (μ = 858.7 m.a.s.l., SD = 327.2) was significantly lower than that of negative individuals (μ = 1212.6 m.a.s.l., SD = 452.7, *p* = 0.0062). In contrast, this was not observed among roe deer infected with *Babesia* sp. EU1 (positive: μ = 720.29 m.a.s.l., SD = 230.55; negative: μ = 872.7 m.a.s.l., SD = 427.42; *p* = 0.218).

### Geographic region

**Figure 2:**
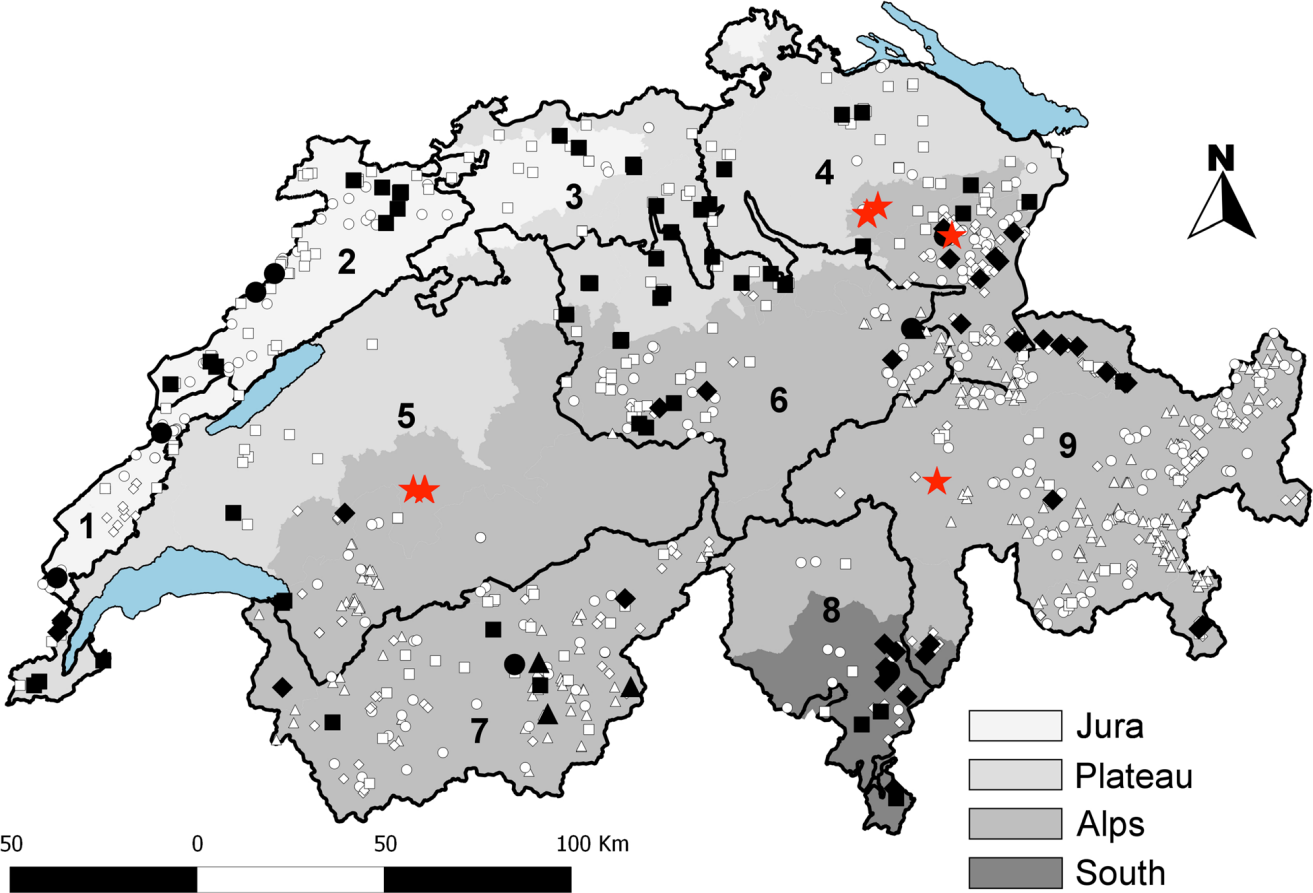
**Map of Switzerland showing the location and infection status of individuals sampled.** Shaded areas represent the four Swiss bioregions, major lakes are in blue. Numbers refer to sampling units: 1) Jura-South, 2) Jura-North, 3) North-West, 4) North-East, 5) Centre-West, 6) Centre-East, 7) South-West, 8) South-Centre, 9) South-East. Black symbols represent animals positive for *Babesia* spp.: Squares: roe deer; Diamonds: red deer; Circles: chamois; Triangles: Alpine ibex. White symbols are individuals that tested negative. Red stars depict the location of chamois positive to *B. capreoli* which were diagnosed post-mortem with clinical babesiosis from 2005 to 2009.

**Figure 3:**
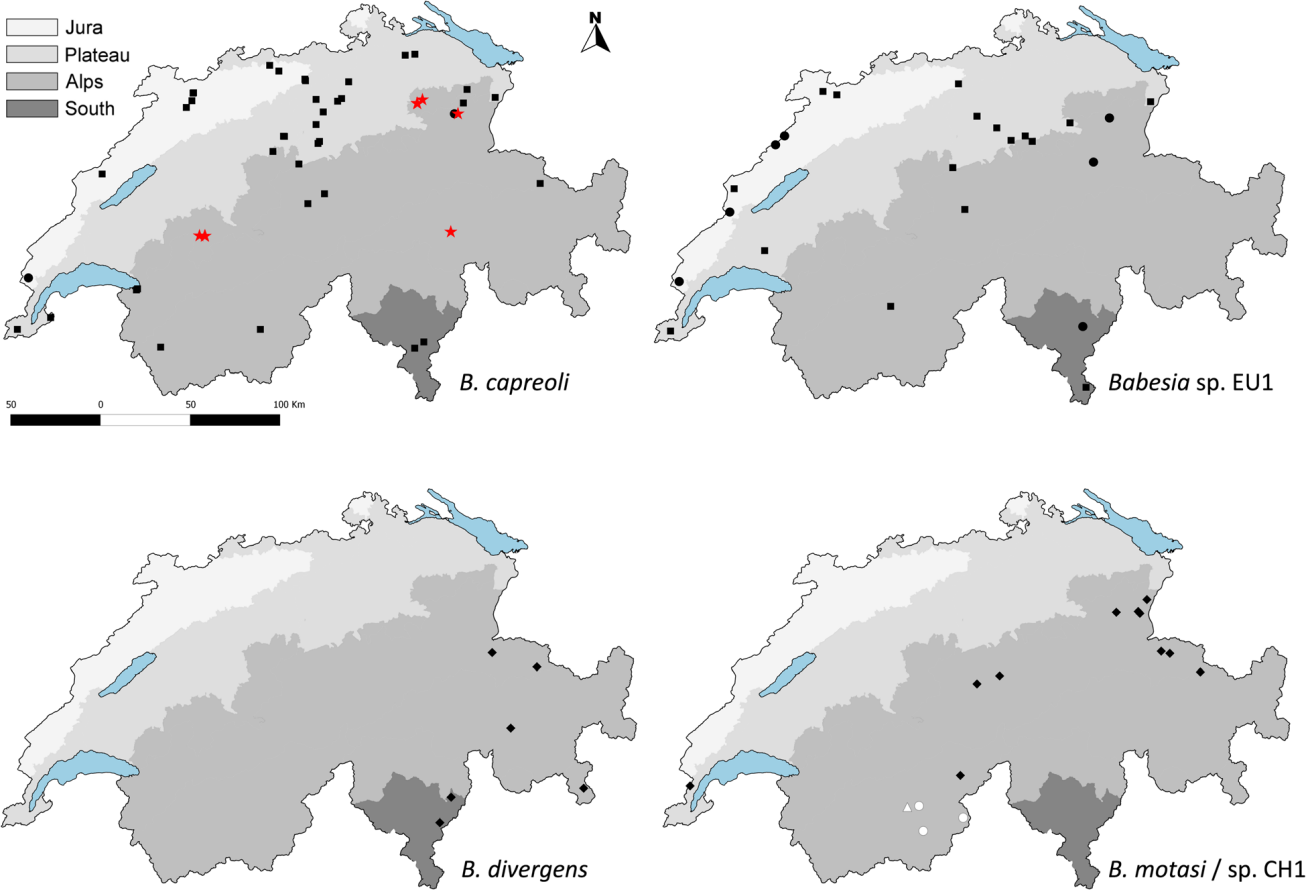
**Maps of Switzerland showing the location of individuals sampled and *Babesia *species identified.** The identity of *Babesia* species tested is given on the bottom right hand side of each map. Shaded areas represent the four Swiss bioregions. Black symbols refer to positive animals: Squares: roe deer; Diamonds: red deer; Circles: chamois; Triangles: Alpine ibex. For the *B. motasi/*sp. CH1 map, individuals positive for *B. motasi* are in dark grey and those positive for *Babesia* sp. CH1 are in black. Negative animals are not mapped. Red stars depict chamois positive to *B. capreoli* which were found with clinical babesiosis from 2005 to 2009.

No differences of prevalence between the different sampling units were observed, neither for *B. capreoli* (*p* = 0.132 to *p =* 1.000) and *Babesia* sp. EU1 (*p* = 0.175 to *p* = 1.000) among roe deer, nor for *B. divergens* (*p* = 0.412 to *p* = 1.000) and *Babesia* sp. CH1 (*p* = 0.125 to *p* = 0.569) among red deer (Figures 2 and 3). Only *B. motasi* was confined to the South-West sampling unit (Figures 2 and 3).

### Cause of death

*Babesia* sp. EU1 infection was significantly more common in roe deer found dead (17.1%, *p* = 0.039) than in those hunted (6.0%). None of the three roe deer and two chamois co-infected with *Babesia* sp. EU1 and *B. capreoli* were found dead. An association between infection and cause of death was not observed for any other host/parasite species combination.

## Discussion

Our study aimed at determining the occurrence and diversity of *Babesia* species in Swiss wild ruminants and at assessing risk factors for infection, in order to better understand the role of roe deer, red deer, Alpine chamois and Alpine ibex in the epidemiology of babesiosis of wildlife, livestock and humans.

### *Babesia divergens*

We report for the first time the identification of *B. divergens* in red deer in Switzerland. Very few studies have focused on the identification of *B. divergens* in red deer in Europe, and in the only other study reporting *B. divergens* in red deer in continental Europe, no attempt was made to confirm the findings by sequencing [14]. Our estimated prevalence for *B. divergens* in red deer (2.6%) is much lower than reported in Ireland and Slovenia (29.0% and 16.7%, respectively [14,28]). However, no *Babesia* species identification could be achieved for as many as 23 of the 40 infected red deer due to poor sequence data. As this occurred only in one roe deer and none of the Caprinae, it seems unlikely that varying sample quality would account for these differences. Additionally, it was shown that serum obtained with the same red deer blood samples (simultaneously collected in different tubes and used for another study [32]) were not more haemolytic than those from other wild ruminant species. Interestingly, Zintl et al. [28] also reported poor sequence quality in many red deer samples, and the reason for this apparently red deer-specific phenomenon remains unclear.

Because of a relatively large proportion of unidentified amplicons, our *Babesia-*specific prevalences in red deer are underestimated. If all of these 23 samples were *B. divergens*, the prevalence (12.6%) would be in a comparative range as in the two other studies. Alternatively, a lower prevalence in the red deer of our study could be explained by the fact that the animals were sampled at higher altitudes than in Slovenia (300-500 m; T. Avsic, personal communication) and Ireland (highest peak at sampling sites: 842 m [39]), suggesting a lower exposure to ticks.

Roe deer have been extensively studied and proposed as a potential host for *B. divergens* but to date, only Welc-Faleciak et al. have identified two roe deer infected with *B. divergens*. However, these two isolates had two unique polymorphic sites in the highly conserved 18S rRNA gene, hence casting doubt as to their proper identity as *B. divergens *[21]. Our present results suggest that *B. divergens* does not occur in roe deer and converge with the observation of Malandrin et al. [11], who concluded from experimental in vitro erythrocyte infection studies that roe deer are not favourable hosts for infection with *B. divergens*. Taken together, these findings suggest that in Switzerland, cervids may not play an important role as primary or mammalian maintenance hosts for *B. divergens*, but an estimate of prevalence in domestic cattle and data on red deer from areas located at lower altitude would be necessary to better address this point.

### *Babesia capreoli*

Of the 38 roe deer infected with *B.* capreoli in our study, 24 were positive by PCR with primers that target a region of the rDNA ITS2 domain known to discriminate between *B. capreoli* and *B. divergens *[27]. To our knowledge, this is the first time that primers have been designed and successfully used to identify samples positive for *B. capreoli.* However, a smaller portion of these samples (*n* = 14) were initially negative with *B. capreoli* primers but matched with 100% identity to *B. capreoli* of roe deer origin [GenBank: AY726009] after sequencing the pan-*Babesia* amplicon. The reason for the apparent lack of sensitivity of the primers is unclear. One putative factor is the modification of the annealing temperature, which had to be increased from 60 °C to 62 °C because of cross-reactivity with *B. divergens* control DNA (not shown). Furthermore, given the limited knowledge about the amplified region of the rDNA ITS2, there could be intra-specific variation within the primer binding sites that could account for this difference.

The relatively high prevalence of *B. capreoli* in roe deer (17.1%), which does not significantly differ from previous studies from Switzerland (26.1%) and Poland (11.9%) [21,30] suggests that roe deer are mammalian maintenance hosts for *B. capreoli*. Red deer however, do not seem to be susceptible to infection.

In the current study, *B. capreoli* was detected in samples from two apparently healthy chamois. Together with earlier data [15,30], our finding of a very low prevalence of *B. capreoli* in Alpine chamois suggests that they are spillover, accidental hosts which mostly succumb to disease upon infection. Indeed, of a total of 317 chamois without reported disease signs, only four were PCR positive (1.3%) while all eight chamois with fatal hemolytic anemia and a marked parasitemia were infected ([15,30]; this study). Furthermore, while diseased animals were thoroughly examined, absence of disease and parasite identification were not definitely confirmed in subclinical infections. Nevertheless, the detection of a few chamois that apparently do not develop disease may be related to host factors such as innate resistance or protective immunity due to exposure early in life [40,41], as well as parasite-specific factors such as differences in the pathogenic potential of various strains [42]. In a former study, *B. capreoli* sequences identified in Alpine chamois that had died of clinical babesiosis [GenBank: EU182596] were identical to those of *B. capreoli* from roe deer, when near full-length 18S rRNA gene sequences were compared to each other.

### *Babesia* sp. EU1

*Babesia* sp. EU1 was identified in roe deer, Alpine chamois and Alpine ibex. Previous studies have shown that this *Babesia* species is common in roe deer [14,17], and given the findings of our study we suggest that roe deer is a mammalian maintenance host for this parasite. Furthermore, to our knowledge, we report for the first time the occurrence of *Babesia* sp. EU1 in Caprinae and document their status as spillover hosts for this parasite. So far, *Babesia sp.* EU1 has never been isolated from a red deer and we provide further evidence that red deer may not be susceptible to infection. *Babesia* spp. are mostly described in the literature as causing infection in only one host. However, *Babesia* sp. EU1, as we document, is able to infect at least three hosts, namely roe deer, Alpine chamois and Alpine ibex. Although our data set does not exclude the possibility of clinical disease due to *Babesia* sp. EU1 in these hosts, there is little evidence to support that contention. However, it is interesting that roe deer found dead were significantly more frequently infected with *Babesia* sp. EU1 than hunted roe deer, raising the possibility that infection with *Babesia* sp. EU1 may have contributed to mortality.

### Concurrent infections with *B. capreoli* and *Babesia* sp. EU1

Concurrent infections of mammalian hosts with multiple *Babesia* species have not been reported to date. However, co-infections of mammalian hosts with tick-borne pathogens of different genera are known to occur, including simultaneous infection with *Babesia* and *Theileria* (reported in cattle) [43] and co-infection with *Babesia* and *Borrelia burgdorferi* (observed in humans) [44]. Similarily, infection of ticks with multiple pathogens has been reported [45-47]. The lack of identification of co-infections with two or more *Babesia* species in mammalian hosts may predominantly result from the applied methods of genetic analysis, which only identify single *Babesia* species from samples. Consequently, multiplex (real-time) PCRs or reverse line blot hybridization should be used to confidently exclude co-infections. Using our PCR-based approach, co-infection status with two *Babesia* species became apparent in five animals. Interestingly, the two apparently healthy Alpine chamois infected with *B. capreoli* were also infected with *Babesia* sp. EU1, and none of the three roe deer with co-infection had been found dead, raising the possibility that co-infection may dampen the pathogenic effect of either *Babesia* species. Indeed, experimental co-infection with *B. divergens* and *Anaplasma phagocytophila* in cattle resulted in markedly reduced hematological abnormalities when compared with animals infected with either pathogen [48]. However, another study suggested that co-infection with two hemoparasites of low virulence can have additive effects and lead to disease, while infection with either one would remain subclinical [43].

### *Babesia motasi*

*Babesia motasi* was identified in three Alpine ibex and one chamois, all originating from the sampling unit South-West. It has never been identified before in wildlife, but the European strain of *B. motasi* – unlike the highly virulent Turkish strain – is a parasite found at low prevalence in sheep and goats in Europe and it does not cause illness [24,49-51]. *Haemaphysialis punctata* is the known vector of *B. motasi *[52] and interestingly in Switzerland this tick species only occurs in the unit South-West [53]. Our findings suggest that Alpine chamois and ibex are hosts of *H. punctata* in southern Switzerland and show that *B. motasi* is able to infect wild Caprinae. The low prevalence at which the parasite is present in these species suggests they are occasional spillover hosts. Given the apparently low pathogenic nature of the parasite, it is expected to pose little risk for domestic or wild ruminant health.

### *Babesia* sp. CH1

*Babesia* sp. CH1 was first discovered in *I. ricinus* ticks feeding on red deer from Switzerland [25] and we show for the first time in this study that the parasite is able to infect red deer. Because the animals sampled were apparently healthy, hunted individuals, there is no indication that *Babesia* sp. CH1 is pathogenic to red deer. Mortality has not been reported in other ruminant species either. Phylogenetically, this parasite is most closely related to *B. odocoilei*, the *Babesia* species of the North-American white-tailed deer, transmitted by *I. scapularis *[54]. The wide spectrum of sequences of this and other similar but not identical *B. odocoilei*-like parasites that have been identified in previous studies [25,28,55] suggests a parasite whose genome may have radiated from a single origin and is well established within the European red deer populations. Given that no other host from our study was positive for this parasite, we hypothesize that red deer is the only susceptible host for this species of *Babesia* among Alpine free-ranging wild ruminants.

### Risk factors for infection

Besides the obvious host-predilection of *Babesia* species identified in this study, age and altitude were found to account for differences in prevalence. Ibex and chamois (Caprinae, prevalence of 2.3%) are less likely than Cervidae (21%) to encounter ticks given the altitude at which they are usually found; it is well reported that tick density decreases with increasing altitude [56,57]. However, our results only partially support the contention that positive animals are more likely to be found at lower altitudes than negative individuals. While *B. capreoli* in roe deer (this study and [30]) and *Babesia* sp. CH1 in red deer are associated with lower altitudinal ranges, it does not seem to be the case for infection with *Babesia* sp. EU1 in roe deer. Nevertheless, this may be due to the occurrence of the parasite in low-lying geographical regions in which the small altitudinal range of the host does not allow any distinction between the location of positive and negative individuals.

Our results suggest that roe deer kids are more often infected with *B. capreoli* or *Babesia* sp. EU1 than are adults. In cattle, it has been shown that calves show few, if any clinical signs of disease upon infection with *B. bovis* and may become persistently infected [40]. In Przewalski horses (*Equus ferus przewalskii*), individuals which are not challenged with equine piroplasms at an early age are unable to cope with an infection in their adult years [41]. These data indicate that exposure early in life determines the outcome of an infection at adult age. Thus, first exposure of roe deer to *B. capreoli* or *Babesia* sp. EU1 at an early age may result in a detectable parasitemia, which may be later reduced to a non-detectable level or cleared by the immune reaction, and lead to a long-lasting protective immunity preventing re-infection.

Except for *B. motasi,* which is confined to the South-West sampling unit, our results do not suggest a particular geographical region as a risk factor for infection. The North-East bioregion, in which the first chamois that died of babesiosis were previously found, did not show a higher prevalence of *B. capreoli.* Although this may be due to a low sample size at local level, it underlines the importance of considering other aspects not measured in our study, such as vector and host occurrence.

### Conclusions

In this study, we have documented the occurrence and diversity of *Babesia* species in a large number of free-ranging ruminants in Switzerland, reporting both previously catalogued and newly discovered parasites in wild ruminants. We show that species of European wild ruminants can be hosts for a range of *Babesia* species; additionally, one individual can be simultaneously infected with more than one species of *Babesia*. Conversely, we also show that certain species of *Babesia* are not specific to one host species.

Furthermore, we propose that cervids are mammalian reservoir hosts for *B. capreoli* (roe deer) and possibly also for *Babesia* sp. EU1 (roe deer) and *Babesia* sp. CH1 (red deer) while their epidemiological role regarding *B. divergens* is more difficult to assess. In contrast, caprids seem to be only spillover or accidental hosts for all *Babesia* species recorded in our study. The occurrence of apparently healthy free-ranging ruminants infected with *B. divergens* or *Babesia* sp. EU1 is an important finding, given the pathogenic potential of these parasites for domestic livestock and/or humans and the wide distribution of their tick vector *I. ricinus *[17].

Finally, the presence of co-infected individuals as well as the higher prevalence of *B. capreoli* and *Babesia* sp. EU1 in juveniles than in adults are interesting from an immunological point of view. First, it converges with former observations that infection early in life does not lead to clinical disease. Second, it questions whether infection with a certain species of *Babesia* may provide cross-protection against the pathogenic effects of a subsequent infection with another *Babesia* species.

## Competing interests

The authors declare that they have no competing interests.

## Authors’ contributions

AOM contributed to the study design and sample collection, performed the molecular and data analyses and drafted the manuscript. AM contributed to the study design and supervised molecular analyses. MPR designed the study, supervised the sample collection and data analysis and drafted the manuscript. All authors critically read and approved the final manuscript. This manuscript is part of the inaugural dissertation of AOM.
